# Genetic Variation Putatively Associated with *Mycobacterium tuberculosis* Resistance to Perchlozone, a New Thiosemicarbazone: Clues from Whole Genome Sequencing and Implications for Treatment of Multidrug-Resistant Tuberculosis

**DOI:** 10.3390/antibiotics9100669

**Published:** 2020-10-03

**Authors:** Igor Mokrousov, Anna Vyazovaya, Gulnora Akhmedova, Natalia Solovieva, Eugeni Turkin, Viacheslav Zhuravlev

**Affiliations:** 1 Laboratory of Molecular Epidemiology and Evolutionary Genetics, St. Petersburg Pasteur Institute, 197101 St. Petersburg, Russia; elmtree2001@mail.ru; 2 Kaliningrad Regional Anti-Tuberculosis Dispensary, 236010 Kaliningrad, Russia; gmakhmedova@mail.ru (G.A.); pt-disp@infomed39.ru (E.T.); 3 St. Petersburg Research Institute of Phthisiopulmonology, 197101 St. Petersburg, Russia; baclab@spbniif.ru (N.S.); jouravlev-slava@mail.ru (V.Z.)

**Keywords:** *Mycobacterium tuberculosis*, drug resistance, perchlozone, thiosemicarbazone, ethionamide, *ethA*, *hadABC*

## Abstract

Perchlozone ([PCZ] 4-thioureido-iminomethylpyridinium perchlorate) is a new thiosemicarbazone approved for the treatment of multidrug-resistant tuberculosis (MDR-TB) in Russia and some other countries. The *ethA* and *hadABC* mutations may confer PCZ resistance. At the same time, *ethA* mutations are known to mediate resistance to ethionamide (ETH) and prothionamide (PTH). We aimed to study the genetic variation underlying *Mycobacterium tuberculosis* resistance to PCZ through whole genome sequencing (WGS) of consecutive isolates recovered during long-term treatment. This prospective study included patients admitted in 2018–2019 to the regional tuberculosis dispensary, Kaliningrad, Russia, whose treatment regimen included PCZ. Multiple *M. tuberculosis* isolates were recovered during PCZ treatment, and the bacterial DNA was subjected to WGS followed by bioinformatics analysis. We identified mutations in the genes putatively associated with PCZ resistance, *ethA*, and *hadA.* The most frequent one was a frameshift *ethA* 106 GA > G (seven of nine patients) and most of the other mutations were also likely present before PCZ treatment. In one patient, a frameshift mutation *ethA* 702 CT > C emerged after six months of PCZ treatment. A frequent presence of cross-resistance mutations to PCZ and ETH/PTH should be taken into consideration when PCZ is included in the treatment regimen of MDR-TB patients.

## 1. Introduction

Emergence and spread of multidrug or extensively drug-resistant (MDR/XDR) *Mycobacterium tuberculosis* strains highlight the importance of the development of new anti-tuberculosis drugs. However, long-term chemotherapy regimens involving such drugs are challenging because they are: (i) expensive, (ii) susceptible to noncompliance, (iii) adverse for a patient, due to side effects, and (iv) favorable to the pathogen, due to the extended time of adaptation. Perchlozone^®^ (4-thioureido-iminomethylpyridinium perchlorate [PCZ]) is a new thiosemicarbazone approved in Russia, along with bedaquiline and delamanid, for treatment of MDR/XDR tuberculosis (TB) [1,2,3,4]. The drug was also approved for use or is in the process of registration in some other countries (https://patentscope.wipo.int/search/en/detail.jsf?docId=WO2011132114&tab=NATIONALPHASE). PCZ is similar to thiacetazone (TAC) and differs from it by the side chain attached to the thiosemicarbazone moiety. Thiacetazone (TAC) was formerly used in combination with isoniazid to treat patients infected with MDR *M. tuberculosis* strains but was removed from the antitubercular chemotherapy due to its secondary toxic effects. TAC is a prodrug that is activated by the flavin-containing monooxygenase EthA to exert its antimycobacterial activity, and mutations in *ethA* are associated with TAC resistance in *M. tuberculosis* [5]. Upon activation, TAC binds to the HadA component of the HadABC dehydratase complex, leading to inhibition of mycolic acid biosynthesis [6,7]. Similarly, PCZ is a prodrug that is activated by EthA and inhibits the HadABC complex. A cross-resistance to TAC and PCZ was shown by in vitro experiments and was mediated by both *ethA* and *hadA* mutations [8]. On the other hand, *hadA* mutations may be acquired by *M. tuberculosis* as a resistance mechanism not only to TAC but also to isoxyl and flavonoids [9]. EthA is known to activate second-line drugs ethionamide (ETH) and prothionamide (PTH), whereas *ethA* or *ethR* mutations were described as one of the ETH/PTH resistance mechanisms [10,11].

In this study, we aimed to gain insight into the molecular basis of PCZ resistance including dynamic changes in *M. tuberculosis* genome during long-term treatment. To this end, we applied next-generation, whole-genome sequencing to the isolates consecutively recovered from patients who received PCZ as part of their chemotherapy regimen.

## 2. Results

In total, 35 isolates were recovered from nine patients who received PCZ (2–6 isolates from each, median four isolates per patient). The first available isolates were resistant to 6 to 11 drugs, including ETH or PTH (Table 1). One patient had MDR-TB, one had pre-XDR-TB and seven had XDR-TB. In addition, two XDR-TB patients (three and five consecutive isolates) who received treatment with new drugs but not PCZ, were included in this study (Table 2). 

Based on the phylogenetically robust markers (single nucleotide polymorphisms (SNP) and deletions), isolates from all patients were assigned to the East-Asian lineage and the Beijing genotype (Table 1; Table 2). The isolates were further subdivided into Beijing B0/W148-cluster (nine patients) and Beijing Central Asia outbreak (CAO) clade (two patients).

The first isolates from nine patients infected with Beijing B0/W148 strain differed in ≥25 SNPs and the first isolates from two patients infected with Beijing CAO strain differed in 29 SNPs (PE and PPE genes, and intergenic regions were excluded from analysis). Accordingly, we conclude that the analyzed strains were not directly epidemiologically linked and did not represent chains of recent transmission.

### 2.1. Mutations: Frequency and Impact

**Figure 1:**
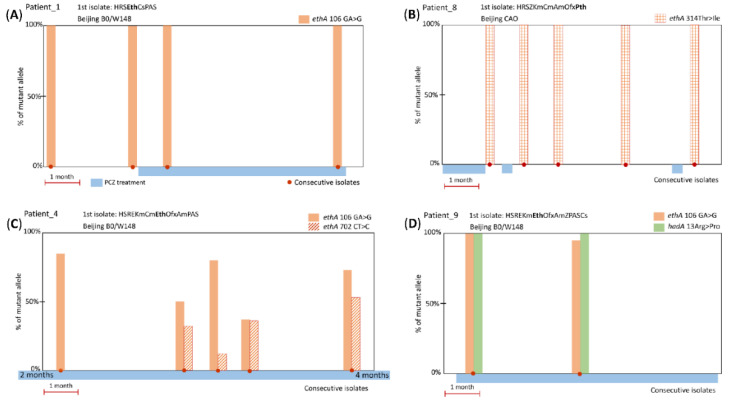
Representative examples of timelines of PCZ treatment, and *ethA* or *hadA* mutations in *M. tuberculosis* isolates. (**A**) Frameshift *ethA* 106 GA > G mutation present in PCZ pre-treatment isolates (5 patients); (**B**) substitution *ethA* 314 ACC > ATC/Thr > Ile (2 patients); (**C**) frameshift *ethA* 702 CT > C emerged during long-term PCZ treatment, in addition to the likely pre-existing *ethA* 106 GA > G (1 patient); (**D**) substitution *hadA* 13 CGG > CCC/Arg > Pro and frameshift *ethA* 106 GA > G (1 patient).

Analysis of genes associated with PCZ resistance (*ethA*, *ethR*, and *hadABC*) identified four mutations: three in *ethA* and one in *hadA* (Table 1; Figure 1). No mutations in the promoter regions of the two genes were detected. The most frequent *ethA* mutation was found for seven of nine PCZ-treated patients and was a frameshift at gene position 106 GA > G (genome position 4,327,363 CT > C). This results in the premature stop codon 62-TAG and considerably abridged 61 amino acid protein. 

The other three mutations were found in one or two cases and two of them were accompanied by the aforementioned mutation *ethA* 106 GA > G (Figure 1). The *ethA* mutation at gene position 702 CT > C (genome position 4,326,770 TA > T) is located inside codon 235 leading to premature stop codon 265-TGA and half-abridged 264 amino acid protein; it was found in one case and was present along with predominant *ethA* 106 GA > G mutation (Figure 1C). 

Two other mutations result in amino acid substitutions: (i) *ethA* 314 ACC > ATC in two cases (Figure 1B) and (ii) *hadA* 13 CGG > CCC in one case (Figure 1D). Both these amino acid changes had low PAM1 scores (Table 3) implying their low probability, which may be interpreted as evidence that these mutations are beneficial to the mycobacterial survival since they were selected and retained.

The *ethA* 106 GA > G mutation was found in the isolates from one of two patients who did not receive PCZ treatment. It was present in all isolates from this patient in 100% reads and all isolates were ETH-resistant (Table 2).

### 2.2. Mutations: Dynamic Changes

Analysis of the consecutive isolates could be helpful in observing gradual development or fluctuation of resistance in response to the drug selective pressure. However, in most cases, all isolates had 100% mutant reads and this may be considered as additional evidence of the previous selection of these mutations during long-term ETH/PTH treatment. In few cases with initial 100% *ethA* frameshift mutation, a wild type allele seemingly reappeared at 5–10% in subsequent isolates (see *ethA* 106 GA > G in Figure 1D). Since a reversion to wild type is improbable, this could be explained by the within-patient heterogeneity of the *M. tuberculosis* strain population and different depth of WGS in different experiments.

A genuine change in the prevalence of mutant and wild type alleles was observed only in one patient. As we hypothesized above, the second mutation *ethA* 702 CT > C emerged after six months of PCZ treatment at 35% frequency and its prevalence in the subsequent isolates fluctuated from 12% to 53% (Figure 1C). These two frameshift *ethA* mutations 106 GA > G and 702 CT > C are separated by ~600 bp and it is not possible to conclude from the WGS data whether they cosegregated on the same read/clone or not. 

## 3. Discussion

A decision to include any additional drug into the complex treatment regimen of MDR/XDR-TB patients should be based on clinical recommendations and informed understanding of possible risks arising, in particular, from cross-resistance to other drugs. To date, only one report—an in vitro study—analyzed the emergence of PCZ resistance [8], and knowledge of the molecular mechanisms of *M. tuberculosis* resistance to this purportedly efficacious drug [3,12] is very limited. Such knowledge is necessary and crucial given that PCZ is included in the list of essential drugs recommended for multidrug-resistant tuberculosis (MDR-TB) chemotherapy by the National Clinical Guidelines endorsed by the National Association of Phthisiatrists in Russia [13]. In turn, the impact of the Russian pool of *M. tuberculosis* strains on the global situation with MDR-TB is undeniable.

A high diversity of mutations scattered throughout the *ethA* gene was identified in the previous studies of the ETH/PTH resistance, where different kinds of mutations were described, including indels or nonsense mutations leading to premature stop codons [11,14,15]. If the EthA activity is lost, ETH is not converted to its active form, which explains the emergence of drug resistance. The lack of dominant *ethA* mutations in ETH-resistant isolates was speculatively attributed to the presence of monooxygenase homologs in *M. tuberculosis* that could protect the cells in case of inactive EthA [16,17]. At the same time, premature stop codons in *ethA* also occurred in susceptible strains and at least two different explanations were proposed, e.g., existence of another ETH-activating enzyme, or a selective pressure by ETH that favors premature stop codon in *ethA* leading to low-levels of resistance [18].

In this study, the first isolates from all nine patients who received PCZ treatment were ETH- or PTH-resistant and the *ethA* mutations were present prior to PCZ treatment in most cases. These mutations could be either acquired during earlier ETH/PTH treatment or a patient could be infected with a resistant strain. The *ethA* 106 GA > G frameshift mutation was present in 100% reads in all pre-PCZ treatment isolates and could be acquired as a mechanism of ETH/PTH resistance (Figure 1A). The substitution *ethA* Thr314Ile was present in 100% reads of the first isolate after 32 days of treatment (Figure 1B) but this timeframe seems too rapid for a mutation not only to emerge but to reach such total dominance in the population. A pre-treatment isolate was not available but it seems more likely that this mutation was present in the infecting strain before PCZ treatment. Only in one patient, the second *ethA* mutation apparently emerged after six months of PCZ treatment (Figure 1C). The observed fluctuation of the presence and prevalence of the frameshift mutations during treatment may speculatively correlate with either heteropopulation (since reversion is improbable) or the existence of one or more enzymes with functional redundancy to EthA.

Concerning *hadA* mutations, TAC and isoxyl are not used in Russia, and it is not known whether patient nine received these drugs (Figure 1D). However, 15 days of PCZ treatment is too short for a mutation not only to emerge but reach 100% frequency and the *hadA* mutation most likely existed before PCZ treatment.

In view of the presence of the particular *ethA* 106 GA > G frameshift mutation in pre-treatment isolates from six patients who did not receive previous PCZ treatment but were all phenotypically resistant to PTH or ETH, we assumed that this mutation emerged as PTH/ETH resistance mutation. We expanded this assumption to three other cases with this mutation. Thus, we conclude that the first isolates from seven of nine patients had this mutation before the PCZ treatment. Two mutations (substitutions *ethA* 314 ACC > ATC/Thr > Ile and *hadA* 13 CGG > CCC/Arg > Pro) were also likely present in isolates before PCZ treatment. Thus, only in one case, a mutation *ethA* 702 CT > C emerged after six months of the PCZ treatment.

We additionally looked for these mutations in the Genome-based *Mycobacterium tuberculosis* Variation (GMTV) database (https://mtb.dobzhanskycenter.org/cgi-bin/beta/main.py#custom/world) [19], defined as a database integrating clinical, epidemiological, and microbiological description with genome variations based on whole genome sequencing (WGS) data of *M. tuberculosis*. While genomic variation information is generated by the database from raw genome data, the accompanying information, such as phenotypic drug susceptibility testing (DST), is provided by original laboratories or studies and cannot be verified.

The *hadA* 13 CGG > CCC mutation was not present in the GMTV database. The most known published *hadA* mutation is in codon 61 [20] and to the best of our knowledge, the described mutation in *hadA13* is novel. On the other hand, all three *ethA* mutations identified in this study were present in GMTV and two of them (314 ACC > ATC and 106 GA > G) were detected in both ETH/PTH-resistant and susceptible isolates (Table 3).

The *ethA* 314 ACC > ATC was detected in 18 Beijing CAO isolates, from Samara in Central Russia (*n* = 17) and St. Petersburg in Northwestern Russia (*n* = 1). This mutation was shown to be specific for the particular subcluster within the CAO clade, which included isolates from Russia and Uzbekistan [21]. We hypothesize that this mutation was selected in the ancestral founding strain of this branch either in response to ETH treatment or as neutral or fitness mutation, in both cases being sufficiently beneficial for mycobacterial survival and transmission.

The *ethA* 106 GA > G was found in the GMTV database in 36 Russian isolates and all belonged to the Beijing B0/W148-cluster (out of 141 B0/W148-cluster isolates present in GMTV).

In contrast to the above two *ethA* mutations detected in both ETH/PTH-resistant and susceptible isolates, the *ethA* 702 CT > C was present in GMTV in six Russian isolates of the Beijing genotype and all five with available DST data were ETH/PTH-resistant. Only three of these six isolates belonged to the Beijing B0/W148 cluster and it may be that this mutation was a homoplastic change independently emerged in unrelated isolates and causatively linked to the ETH/PTH resistance. Otherwise, the information on phenotypic resistance to ETH/PTH should be regarded with caution since DST for these two drugs is known to be problematic and analysis of *inhA*, *ethA*, and *ethR* is recommended as a reference method by WHO [22]. This situation highlights a controversy between the above-cited articles (where DST is considered a gold standard) and WHO guidelines as to what approach to the ETH resistance testing should be adopted as the primary one.

In conclusion, the frequent presence of cross-resistance mutations to both PCZ and ETH/PTH presents an especially worrisome finding of this study (although on a positive side one may note that the emergence of PCZ resistance during PCZ treatment was rare). This situation raises a major concern with regard to the non-efficiency of PCZ in the treatment of a significant number of MDR-TB cases whose isolates may be additionally resistant to ETH/PTH. In view of the high and increasing burden of MDR-TB in Russia, ETH and PTH are frequently used to treat such patients. The ETH/PTH regimen can take at least 18–24 months as recommended by national and international guidelines [13,22] and ETH/PTH resistance can emerge quite frequently in clinical isolates, especially in MDR isolates [23].

The treatment regimen of the XDR-TB patients includes at least six effective drugs and the presence of PCZ resistance mutations may not be necessarily associated with treatment failure. However, the inclusion of the non-effective additional drug in the treatment regimen is impractical and may be adverse for a patient’s health. To adequately assess the association of the identified mutations with PCZ resistance, an implementation of the phenotypic PCZ susceptibility testing is urgently needed. A large prospective study of the diverse *M. tuberculosis* collection is warranted to formulate the recommendations for optimal use of PCZ, taking into consideration possible ETH/PTH resistance of the isolates.

## 4. Materials and Methods 

The study was performed in the Kaliningrad region of Russia. The TB incidence in Kaliningrad decreased from 134/100,000 in 2006 to 50.6/100,000 in 2015. Nevertheless, this latter figure exceeds both a mean figure for Northwest Russia (40.7/100,000) and the country as a whole (57.7/100,000). The rate of primary MDR-TB in the region increased from 23.9% in 2010 to 30.5% in 2015 (http://mednet.ru/). This region is located in the westernmost part of the Russian Federation and makes an exclave on the Baltic Sea inside the European Union separated from the mainland Russia by Lithuania and Poland. Virtually closed from foreign contact from the 1950s until 1991, Kaliningrad today is a busy Russian–European crossroad that has an impact on the epidemiological situation of communicable diseases not only in Russia but also in northern Europe [24].

This prospective study included 11 patients admitted to the regional tuberculosis dispensary of the Kaliningrad region in 2018–2019. Nine patients received PCZ as a part of chemotherapy. No links between patients were identified based on the standard epidemiological investigation. All samples used in this study were coded and lacked personal information about the patients, especially names and addresses, to maintain their anonymity. Patient-related data were obtained anonymously, and no individual patient information is disclosed in this article. The study was approved by the Ethics Committee of the Research Institute of Phthisiopulmonology (protocol 31.2 of February 27, 2017).

*M. tuberculosis* isolates were recovered at the 1–5 month intervals. *M. tuberculosis* DST for first- and second-line drugs was performed using a modified proportion method on the Middlebrook 7H9 liquid culture and Bactec MGIT 960 system (Becton Dickinson, Sparks, Md.) according to the manufacturer’s instructions and national guidelines (order #951 of 29 December 2014 “On the approval of guidelines for improving the diagnosis and treatment of respiratory tuberculosis”, Ministry Healthcare of the Russian Federation). The critical drug concentrations used were 1.0 μg/mL for streptomycin, 0.1 μg/mL for isoniazid, 5.0 μg/mL for ethambutol, 1.0 μg/mL for rifampicin, 100 μg/mL for pyrazinamide, 1.0 μg/mL for amikacin, 2.5 μg/mL for capreomycin, 2.0 μg/mL for ofloxacin, 5 μg/mL for ethionamide [13]. The laboratory is externally quality assured by the System for External Quality Assessment “Center for External Quality Control of Clinical Laboratory Research” (Moscow, Russia). 

A limitation of this study was that the phenotypic PCZ resistance data was not available for the studied isolates since no critical concentrations have been approved and PCZ susceptibility testing is not yet implemented. Based on the previous reports on PCZ and TAC resistance, we assumed mutations in *ethA, ethR,* and *haABC* as a genetic proxy of phenotypic resistance to PCZ.

DNA was extracted from bacterial cultures grown on the Loewenstein–Jensen medium using a CTAB-based method as previously described [25].

Whole genome sequencing was performed at the MiSeq or NextSeq 500 platforms (Illumina). DNA libraries were prepared using ultrasound DNA fragmentation and NEBNext Ultra DNA Library Prep Kit for Illumina (New England Biolabs). Data for the *M. tuberculosis* sequenced genomes were deposited in the NCBI Sequence Read Archive (project numbers PRJNA525341 and PRJNA635788). The fastq files were aligned to the complete genome of the reference strain H37Rv (NC_00962.3) using Geneious 9 program (Biomatters, New Zealand) and additionally checked with PhyReSE online tool (https://bioinf.fz-borstel.de/mchips/phyresse/). The mean read length was 150 bp for NextSeq 500 data and 250 bp for MiSeq data. An average genome coverage for the analyzed genome regions was x103 for the NextSeq 500 data and x31 for the MiSeq data.

*M. tuberculosis* isolates (genomes) were assigned to the Beijing genotype and its specific subtypes based on the assessment of the previously described SNP markers and genomic deletions [26,27,28,29,30]. The short sequencing reads (fastq files) were subjected to in silico spoligotyping using TGS-TB online tool at https://gph.niid.go.jp/tgs-tb/ [31] to deduce their spoligoprofile.

## Figures and Tables

**Table 1:** Summary of enrolled tuberculosis patients and infecting *M. tuberculosis* isolates for patients treated with perchlozone.

Pt.	Phenotypic Resistance Profile of the First Isolate	Treatment Scheme	Possible PCZ Resistance Mutations: Position in the Reference Genome, Position in Gene, Comment	*M. tuberculosis* Genotype
1.	HRSEthCsPAS	ZELfxAmLzdBq	4327363 CT > C, *ethA* 106 GA > G, pre-existed	Beijing B0/W148
2.	HRSEKmCmEthOfxCsAmPAS	ZMfxLzdPczBq	4327363 CT > C, *ethA* 106 GA > G, pre-existed	Beijing B0/W148
3.	HRSEOfxEthPAS	CmZMfxTrdPczBq	4327363 CT > C, *ethA* 106 GA > G, pre-existed	Beijing B0/W148
4.	HSREKmCmEthOfxAmPAS	ZCsCmMfxLfxPczBq	4327363 CT > C, *ethA* 106 GA > G, pre-existed 4326770 TA > T, *ethA* 702 CT > C, emerged	Beijing B0/W148
5.	HRSZEKmCmOfxCsPth	AmZLfxPASPczBq	4327363 CT > C, *ethA* 106 GA > G, pre-existed	Beijing B0/W148
7.	HRSEKmCmAmPthOfxPASCs	ZLfxTrdPczBq	4326533 G > A, *ethA* 314 ACC > ATC/Thr > Ile, likely pre-existed	Beijing CAO
8.	HRSZKmCmAmOfxPth	ECsLfxLzdPczBq	4326533 G > A, *ethA* 314 ACC > ATC/Thr > Ile, likely pre-existed	Beijing CAO
9.	HSREKmEthOfxAmZPASCs	ZLfxClzPczBq	4327363 CT > C, *ethA* 106 GA > G, pre-existed 731967 G > C, *hadA* 13 CGG > CCC/Arg > Pro, pre-existed	Beijing B0/W148
11.	HRSZEEthOfxCmPAS	AmCsLfxPczBq	4327363 CT > C, *ethA* 106 GA > G, pre-existed	Beijing B0/W148

Abbreviations. FCT: fibrocavernous TB, pulmonary TB: PTB. CAO: Central Asia outbreak. Antibiotics: H—isoniazid, R—rifampin, S—streptomycin, E—ethambutol, Lfx—levofloxacin, Mfx—moxifloxacin, Ofx—ofloxacin, Cs—cycloserine, Am—amikacin, Km—kanamycin, Cm—capreomycin, PAS—Para-aminosalicylic acid, Z—pyrazinamide, Pcz—perchlozone, Bq—bedaquiline, Clz—clofazimine, Lz—linezolid, Trd—Terizidone, Eth—ethionamide, Pth—prothionamide.

**Table 2:** Summary of enrolled tuberculosis patients and infecting *M. tuberculosis* isolates for patients not treated with perchlozone.

Pt	Phenotypic Resistance Profile of the First Isolate	Treatment Scheme	Possible PCZ Resistance Mutations: Position in the Reference Genome, Position in Gene, Comment	*M. tuberculosis* Genotype
6.	HRSEKmOfx	ZCmMfxTrdPthBq	4327363 CT > C, *ethA* 106 GA > G, pre-existed	Beijing B0/W148
10.	HSREKmEtoOfxCs	AmZLfxPASBq	-	Beijing B0/W148

Abbreviations. FCT: fibrocavernous TB, pulmonary TB: PTB. CAO: Central Asia outbreak. Antibiotics: H—isoniazid, R—rifampin, S—streptomycin, E—ethambutol, Lfx—levofloxacin, Mfx—moxifloxacin, Ofx—ofloxacin, Cs—cycloserine, Am—amikacin, Km—kanamycin, Cm—capreomycin, PAS—Para-aminosalicylic acid, Z—pyrazinamide, Pcz—perchlozone, Bq—bedaquiline, Clz—clofazimine, Lz—linezolid, Trd—Terizidone, Eth—ethionamide, Pth—prothionamide.

**Table 3:** Mutations in genes putatively associated with PCZ resistance in *M. tuberculosis* isolates recovered from PCZ-treated patients.

Mutation, Position in Gene, Codon (Amino Acid Change)	Possible Impact	This Study, Number of Patients	Frequency, %, Range of Mutant Short Sequencing Reads in Different Isolates	Presence in GMTV Database*: Number of Isolates and Details
*ethA* 106 GA > G	Deletion and frameshift: premature stop codon 62-TAG and considerably abridged 61 amino acid protein	7	35–100	36 isolates from Russia, Samara (*n* = 31) and St. Petersburg (*n* = 5); 14 ETH/PTH resistant to and 12 were susceptible; all belong to the Beijing B0/W148-cluster (out of 141 B0/W148 isolates present in GMTV)
*ethA* 702 CT > C	Deletion and frameshift: premature stop codon 265-TGA and half-abridged 264 amino acid protein	1	12–53	6 isolates from Samara (*n* = 3) and St. Petersburg (*n* = 3); 5 ETH/PTH resistant; all belong to the Beijing genotype, 3 of 6 Beijing B0/W148-cluster
*ethA* 314 ACC > ATC (Thr > Ile)	Substitution with low PAM1 score** (=3) and likely impact on the protein structure	2	100	18 isolates, originating from the Samara region in central Russia (*n* = 17) and St. Petersburg (*n* = 1); 4 were resistant to ETH/PTH and 7 were susceptible. All 18 belong to the Beijing CAO cluster (out of 65 CAO isolates present in GMTV)
*hadA* 13 CGG > CCC (Arg > Pro)	Substitution with low PAM1 score (=5) and likely impact on the protein structure	1	100	Not present

* Genome-based *Mycobacterium tuberculosis* Variation Database (GMTV): https://mtb.dobzhanskycenter.org/cgi-bin/beta/main.py#custom/world. ** PAM1—Point Accepted Mutation 1; it gives the probability (multiplied with 10,000) for the particular amino acid exchange to occur, given that 1% of the amino acids are changed.

## References

[1] Vinogradova T.I., Aleksandrova A.E., Antonenkova E.V., Elokhina V.N., Nakhmanovich A.S. (1999). Design and study of new agents having antitubercular activity: The original compound perchlosone as a potent agent of etiotropic therapy for tuberculosis. Probl. Tuberk..

[2] Yablonskiy P.K., Vinogradova T.I., Levashev Y.N., Pavlova M.V., Zilber E.K., Starshinova A.A., Sapozhnikova N.V., Chernokhaeva I.V., Archakova L.I., Zabolotnykh N.V. (2016). Preclinical and clinical trials of the new tuberculosis drug perchlozon. Ter Arkh..

[3] Churilov L., Korzhikov-Vlakh V., Sinitsyna E., Polyakov D., Darashkevich O., Poida M., Platonova G., Vinogradova T., Utekhin V., Zabolotnykh N. (2018). Enhanced delivery of 4-thioureidoiminomethylpyridinium perchlorate in tuberculosis models with IgG functionalized poly(Lactic acid)-based particles. Pharmaceutics.

[4] Gushchin A.S., Vinogradova T.I., Yablonskiy P.K., Batyunin G.A., Zabolotnyh N.V., Vasilieva S.N., Maligin A.V. (2017). Tuberculosis Drug Based on 4-Thioureido-Iminomethylpyridinium Perchlorate: Method of Preparation and Treatment. U.S. Patent.

[5] Dover L.G., Alahari A., Gratraud P., Gomes J.M., Bhowruth V., Reynolds R.C., Besra G.S., Kremer L. (2007). EthA, a common activator of thiocarbamide-containing drugs acting on different mycobacterial targets. Antimicrob. Agents Chemother..

[6] Halloum I., Viljoen A., Khanna V., Craig D., Bouchier C., Brosch R., Coxon G., Kremer L. (2017). Resistance to thiacetazone derivatives active against Mycobacterium abscessus involves mutations in the MmpL5 transcriptional repressor MAB_4384. Antimicrob. Agents Chemother..

[7] Belardinelli J.M., Morbidoni H.R. (2012). Mutations in the essential FAS II-hydroxyacylACP dehydratase complex confer resistance to thiacetazone in Mycobacterium tuberculosis and Mycobacterium kansasii. Mol. Microbiol..

[8] Gopal P., Dick T. (2015). The new tuberculosis drug Perchlozone^®^ shows cross-resistance with thiacetazone. Int. J. Antimicrob. Agents.

[9] Dong Y., Qiu X., Shaw N., Xu Y., Sun Y., Li X., Li J., Rao Z. (2015). Molecular basis for the inhibition of β-hydroxyacyl-ACP dehydratase HadAB complex from Mycobacterium tuberculosis by flavonoid inhibitors. Protein Cell..

[10] Islam M.M., Tan Y., Hameed H.M.A., Liu Z., Chhotaray C., Liu Y., Lu Z., Cai X., Tang Y., Gao Y. (2019). Detection of novel mutations associated with independent resistance and cross-resistance to isoniazid and prothionamide in Mycobacterium tuberculosis clinical isolates. Clin. Microbiol. Infect..

[11] De Welzen L., Eldholm V., Maharaj K., Manson A.L., Earl A.M., Pym A.M. (2017). Whole-transcriptome and -genome analysis of extensively drug-resistant Mycobacterium tuberculosis clinical Iisolates identifies downregulation of ethA as a mechanism of ethionamide resistance. Antimicrob. Agents Chemother..

[12] Chernokhaeva I., Pavlova M., Starshinova A., Sapozhnikova N., Belaeva E., Zhuravlev V., Archakova L., Yablonskii P., Starshinova A. (2015). Therapy of pulmonary tuberculosis with multidrug-resistant Mycobacterium tuberculosis using tioureidoiminomethylpyridinium perchlorate (Perchlozon). Int. J. Tech. Res. Appl..

[13] Yablonsky P.K. (2015). Phthisiatry National Clinical Guidelines.

[14] Tan Y., Su B., Zheng H., Song Y., Wang Y., Pang Y. (2017). Molecular characterization of prothionamide-resistant Mycobacterium tuberculosis isolates in Southern China. Front. Microbiol..

[15] Rueda J., Realpe T., Mejia G.I., Zapata E., Rozo J.C., Ferro B.E., Robledo J. (2015). Genotypic analysis of genes associated with independent resistance and cross-resistance to isoniazid and ethionamide in Mycobacterium tuberculosis clinical isolates. Antimicrob. Agents Chemother..

[16] Morlock G.P., Metchock B., Sikes D., Crawford J.T., Cooksey R.C. (2003). ethA, inhA, and katG loci of ethionamide-resistant clinical Mycobacterium tuberculosis isolates. Antimicrob. Agents. Chemother..

[17] Vilchèze C., Jacobs W.R. (2014). Resistance to isoniazid and ethionamide in Mycobacterium tuberculosis: Genes, mutations, and causalities. Microbiol. Spectr..

[18] Johnsen C.H., Clausen P.T.L.C., Aarestrup F.M., Lund O. (2019). Improved resistance prediction in Mycobacterium tuberculosis by better handling of insertions and deletions, premature stop codons, and filtering of non-informative sites. Front. Microbiol..

[19] Chernyaeva E.N., Shulgina M.V., Rotkevich M.S., Dobrynin P.V., Simonov S.A., Shitikov E.A., Ischenko D.S., Karpova I.Y., Kostryukova E.S., Ilina E.N. (2014). Genome-wide Mycobacterium tuberculosis variation (GMTV) database: A new tool for integrating sequence variations and epidemiology. Bmc Genom..

[20] Grzegorzewicz A.E., Eynard N., Quémard A., North E.J., Margolis A., Lindenberger J.J., Jones V., Korduláková J., Brennan P.J., Lee R.E. (2015). Covalent modification of the Mycobacterium tuberculosis FAS-II dehydratase by Isoxyl and Thiacetazone. ACS Infect. Dis..

[21] Köser C. (2015). Implications of the Genetic Diversity within MTBC and *M. canettii* for the Development of New DST Assays. http://www.stoptb.org/wg/new_diagnostics/assets/documents/NDWD_AnnMtg2015_02-03_Claudio_KOSER.pdf.

[22] World Health Organization (2019). WHO Consolidated Guidelines on Drug-Resistant Tuberculosis Treatment.

[23] Ismail N.A., Mvusi L., Nanoo A., Dreyer A., Omar S.V., Babatunde S., Molebatsi T., van der Walt M., Adelekan A., Deyde V. (2018). Prevalence of drug-resistant tuberculosis and imputed burden in South Africa: A national and sub-national cross-sectional survey. Lancet Infect. Dis..

[24] Vyazovaya A.A., Akhmedova G.M., Solovieva N.S., Gerasimova A.A., Starkova D.A., Turkin E.N., Zhuravlev VYu Narvskaya O.V., Mokrousov I.V. (2017). Molecular epidemiology of tuberculosis in the Kaliningrad region of Russia: 10 years after. Russ. J. Infect. Immun..

[25] van Embden J.D., Cave M.D., Crawford J.T., Dale J.W., Eisenach K.D., Gicquel B., Hermans P., Martin C., McAdam R., Shinnick T.M. (1993). Strain identification of Mycobacterium tuberculosis by DNA fingerprinting: Recommendations for a standardized methodology. J. Clin. Microbiol..

[26] Tsolaki A.G., Gagneux S., Pym A.S., Goguet de la Salmoniere Y.O., Kreiswirth B.N., Van Soolingen D., Small P.M. (2005). Genomic deletions classify the Beijing/W strains as a distinct genetic lineage of Mycobacterium tuberculosis. J. Clin. Microbiol..

[27] Mokrousov I., Narvskaya O., Vyazovaya A., Otten T., Jiao W.W., Gomes L.L., Suffys P.N., Shen A.D., Vishnevsky B. (2012). Russian “successful” clone B0/W148 of Mycobacterium tuberculosis Beijing genotype: A multiplex PCR assay for rapid detection and global screening. J. Clin. Microbiol..

[28] Shitikov E., Kolchenko S., Mokrousov I., Bespyatykh J., Ischenko D., Ilina E., Govorun V. (2017). Evolutionary pathway analysis and unified classification of East Asian lineage of Mycobacterium tuberculosis. Sci. Rep..

[29] Mokrousov I., Chernyaeva E., Vyazovaya A., Skiba Y., Solovieva N., Valcheva V., Levina K., Malakhova N., Jiao W.W., Gomes L.L. (2018). Rapid assay for detection of the epidemiologically important central Asian/Russian strain of the Mycobacterium tuberculosis Beijing genotype. J. Clin. Microbiol..

[30] Shitikov E., Vyazovaya A., Malakhova M., Guliaev A., Bespyatykh J., Proshina E., Pasechnik O., Mokrousov I. (2019). Simple assay for detection of the central Asia outbreak clade of the Mycobacterium tuberculosis Beijing genotype. J. Clin. Microbiol..

[31] Sekizuka T., Yamashita A., Murase Y., Iwamoto T., Mitarai S., Kato S., Kuroda M. (2015). TGS-TB: Total genotyping solution for Mycobacterium tuberculosis using short-read whole-genome sequencing. PLoS ONE.

